# Epigenetic differences between naïve and primed pluripotent stem cells

**DOI:** 10.1007/s00018-017-2703-x

**Published:** 2017-11-13

**Authors:** Saori Takahashi, Shin Kobayashi, Ichiro Hiratani

**Affiliations:** 1 Laboratory for Developmental Epigenetics, RIKEN Center for Developmental Biology, 2-2-3 Minatojima-minamimachi, Chuo-ku, Kobe, 650-0047 Japan; 2 Molecular Profiling Research Center for Drug Discovery, National Institute of Advanced Industrial Science and Technology, 2-4-7 Aomi, Koutou-ku, Tokyo, 135-0064 Japan; 3 Department of Epigenetics, Medical Research Institute, Tokyo Medical and Dental University, 1-5-45 Yushima, Bunkyo-ku, Tokyo, 113-8510 Japan

**Keywords:** Epigenetics, Naïve and primed pluripotency, Embryonic stem cells (ESCs), Epiblast-derived stem cells (EpiSCs), X-chromosome inactivation (XCI), Three-dimensional (3D) genome organization

## Abstract

It has been 8 years since the concept of naïve and primed pluripotent stem cell states was first proposed. Both are states of pluripotency, but exhibit slightly different properties. The naïve state represents the cellular state of the preimplantation mouse blastocyst inner cell mass, while the primed state is representative of the post-implantation epiblast cells. These two cell types exhibit clearly distinct developmental potential, as evidenced by the fact that naïve cells are able to contribute to blastocyst chimeras, while primed cells cannot. However, the epigenetic differences that underlie the distinct developmental potential of these cell types remain unclear, which is rather surprising given the large amount of active investigation over the years. Elucidating such epigenetic differences should lead to a better understanding of the fundamental properties of these states of pluripotency and the means by which the naïve-to-primed transition occurs, which may provide insights into the essence of stem cell commitment.

## Introduction

In 2007, a new type of stem cells, the epiblast-derived stem cells (EpiSCs), was isolated from the post-implantation epiblast in mice [1, 2]. These pluripotent cells possess features that distinguish them from mouse embryonic stem cells (mESCs). It was an exciting time for the field of stem cell research, as many groups were following up and building on the reprogramming experiments described in the first report of the isolation of induced pluripotent stem cells (iPSCs) via forced expression of four transcription factors in somatic cells [3, 4]. Since the discovery of EpiSCs and techniques for deriving iPSCs occurred within a short time frame, the possibility of multiple stable and metastable pluripotent states soon emerged, eventually leading to the proposal of naïve and primed pluripotent states representing the distinct cellular identities of pre- and post-implantation epiblast cells, respectively [5]. This in turn raised the question of how cells could transition between naïve and primed states, with particular interest in the ‘reverse’ transition from primed-to-naïve state (i.e., reprogramming).

Differences between naïve and primed pluripotency have been extensively studied, from culture conditions and functional capacities to gene expression profiles and chromatin modification states, as outlined in an excellent recent review by Weinberger et al. [6]. However, many of these properties are not readily observable without undergoing days or even weeks of experimental procedures and/or treatments. Examples of such not immediately detectable properties include the long-term dependence of primed (but not naïve) cells on Activin and FGF signaling, and the inability of primed (but not naïve) cells to contribute to blastocyst chimera formation. This raises the question of whether there is a decisive intrinsic difference that demarcates naïve from primed cells that is also readily observable. Epigenetic signatures on their chromatin represent an attractive candidate for study, as these should form the basis for their respective cell-type-specific gene expression programmes and differences in their functional capacities. However, a fuller understanding of these epigenetic differences remains a distant goal. In this review, we discuss the state of the science with regard to pluripotent cell epigenetics and look ahead to potential areas of investigation that might provide new breakthroughs.

We should also note here that there may in fact be a continuum of intermediate states between naïve and primed states in vivo [7]. However, not all such states have been captured in vitro and moreover, even for intermediate states reported to date, their epigenetic status has not been thoroughly analyzed. For this reason, we mainly focus on the naïve and primed states in this article, and subsequently address one intermediate state, the formative pluripotent state, in depth [8].

## Brief historical overview of naïve and primed pluripotency

In 1981, it was first reported that mouse embryonic stem cells (mESCs) had been established from the inner cell mass (ICM) of late blastocysts [9, 10]. At the time, teratocarcinoma formation after transplantation into immunodeficient mice was the primary test of cellular pluripotency. In 1984, mESCs were shown to contribute to the formation of chimeric mice after injection into the blastocyst, and this assay then became the gold standard test of pluripotency and remained so for many years [11]. Later, in 1998, human ESCs (hESCs) were isolated from the ICM, and it became clear that the culture conditions used for growing hESCs were distinct from that used for mESCs; the requirement or lack thereof for Activin A and FGF2 being examples of these differences [12–14]. Moreover, many female hESC lines exhibited an inactive X chromosome (Xi), which suggested that the epigenetic signatures of hESCs were distinct from those of mESCs [15]. Then, in 2007, it was reported that when the post-implantation epiblasts of E5.5 mouse embryos were cultured under the same conditions as those used for hESCs, a new type of stem cell could be isolated; these were named EpiSCs [1, 2]. Indeed, mouse EpiSCs (mEpiSCs) exhibited characteristics similar to hESCs in various aspects such as X-inactivation and poor survival after single-cell suspension. Unlike mESCs, mEpiSCs rarely contributed to chimeric mice [1, 2], which suggested that their developmental potentials are distinct, with mEpiSCs representing a more restricted state.

In a parallel development to this line of work, mouse iPSCs (miPSCs) were established in 2006 [3]. The iPSCs initially reported by Yamanaka and colleagues [3] were considered to be partially reprogrammed because they formed teratomas but failed to contribute to chimeric mice. It did not take long, however, to establish iPSCs capable of contributing to chimera formation [16–18]. Interestingly, in these more fully reprogrammed ‘standard’ iPSC clones, the Xi was reactivated [18]. In contrast, partially reprogrammed iPSC clones that formed teratomas but failed to contribute to chimeric mice maintained the Xi, suggesting that they had epigenetic signatures distinct from the standard iPSCs [18]. It should be noted that no iPSC is completely reprogrammed epigenetically: for instance, iPSCs retain residual DNA methylation patterns of parental somatic cells, while mESCs generated via somatic cell nuclear transfer exhibit a more complete erasure, resembling that in conventional mESCs [19, 20]. As for mEpiSCs, it was soon discovered that mEpiSCs exhibit an Xi [21]. Thus, in both differentiation and reprogramming, the Xi state appears to be tightly associated with the differentiation state of cells and their developmental potential [22–25].

Given these findings, it was proposed that there exist two stem cell states with distinct epigenetic signatures, which the authors named naïve and primed pluripotent states [5]. Naïve mESCs are derived from the ICM of the preimplantation blastocyst and are cultured in serum/LIF or 2i/LIF medium (two inhibitors (i) for MEK and GSK3 along with leukemia inhibitory factor LIF), in which they show round, dome-shaped cell colony morphology [6]. Primed mEpiSCs are derived from the post-implantation epiblast and require Activin and FGF signaling [1, 2]. Unlike mESCs, mEpiSC colonies grow as a monolayer and are morphologically similar to hESC colonies [1]. Moreover, mEpiSCs cannot be dissociated down to a single-cell suspension; if this is attempted the cells will undergo apoptosis in a manner dependent on the Rho-associated, coiled-coil containing protein kinase (ROCK) pathway [26]. Both naïve and primed cells utilize the Oct4/Sox2/Nanog transcription factor network, although their genome-wide binding profiles are distinct and their target genes differ slightly between naïve and primed states [6, 27]. Thus, the gene expression profiles of naïve and primed cells are similar, but distinct.

## Naïve vs. primed: what could be the most critical epigenetic difference?

Naïve mESCs can be differentiated to a primed mEpiSC-like cell state by culturing mESCs in mEpiSC culture conditions [21, 28]; however, conversion in the opposite direction is challenging, which suggests the presence of some form of epigenetic barrier [21, 29, 30]. One obvious manifestation of developmental potential is the transcriptome, which is similar but distinct in mESCs and mEpiSCs [1, 2]. What could be the epigenetic difference underlying their distinct developmental potentials? In other words, what could be the most critical difference(s) in their chromatin structure?

In a narrow sense, major epigenetic marks as we know them today can be subdivided into two types: histone modifications and DNA methylation. Histone modification patterns are distinct between naïve and primed cells [1]. However, it is difficult to describe all of the differences in histone modifications concisely and pinpoint the ones that are critical. Moreover, it remains controversial whether histone modifications are a cause or a consequence of gene expression patterns. Distinct histone modification patterns on gene promoters may simply reflect their distinct transcription states [31–33].

Differences in enhancer histone modifications between naïve and primed cells have also been reported [34], and enhancer usage in these cells differs, even for genes expressed in both states [34]. A good example is Oct4 enhancer usage, in which the distal enhancer (DE) is preferentially utilized in the naïve state, whereas the proximal enhancer (PE) is primarily used in the primed state [1, 35]. This distinction implies differences in long-range chromatin interactions, which may contribute to the local three-dimensional (3D) genome organization.

DNA methylation also varies across the naïve and primed states; it has been reported that the genomic DNA of naïve mESCs is generally hypomethylated, whereas in primed mEpiSCs it is hypermethylated [6, 36, 37]. In fact, naïve mESCs cultured in 2i/LIF medium in vitro exhibit a widespread loss of DNA methylation, including genomic imprints [38]. In ‘less naïve’ mESCs grown in serum/LIF medium, the DNA methylation level is clearly higher than that in naïve mESCs grown in 2i/LIF, although not as high as in mEpiSCs [39, 40]. Thus, DNA methylation state is one clear example of epigenetic differences between mESCs and mEpiSCs. Surprisingly however, this difference is observed only in vitro and not in the in vivo counterparts of these cell types; it has been shown that the mouse epiblast cells are globally DNA hypomethylated, both pre- and post-implantation [41]. Thus, any epigenetic difference between mESCs and mEpiSCs may not readily translate to a difference between naïve and primed states in vivo. It is also important to note that a cause–effect relationship has not been established. DNA methylation is enriched in repressed genes in mEpiSCs, but this could merely reflect gene repression [42].

Thus, while various epigenetic marks have been analyzed, it remains to be determined exactly which epigenetic difference demarcates naïve and primed states. Moreover, identifying chromatin modification differences is only the beginning, as the daunting task of addressing whether such differences are a cause or a consequence of their distinct transcription patterns also remains.

## Naïve vs. primed: signs of X-chromosome inactivation exist only in the latter

While our initial intent was to widely cover and summarize the current knowledge on the epigenetic differences between naïve and primed states, we came to realize that no crucial epigenetic difference between naïve and primed cells in the form of chromatin modifications has been identified. In the context of embryonic development (the context for which Conrad Waddington first coined the term ‘epigenetics’), mEpiSCs are clearly more advanced than mESCs [43, 44]. Moreover, mESCs contribute to chimeric mice, while mEpiSCs do so only rarely. This strongly suggests differences in their developmental potential, and thus some form of epigenetic signature that is distinct between these two cell types.

**Fig. 1:**
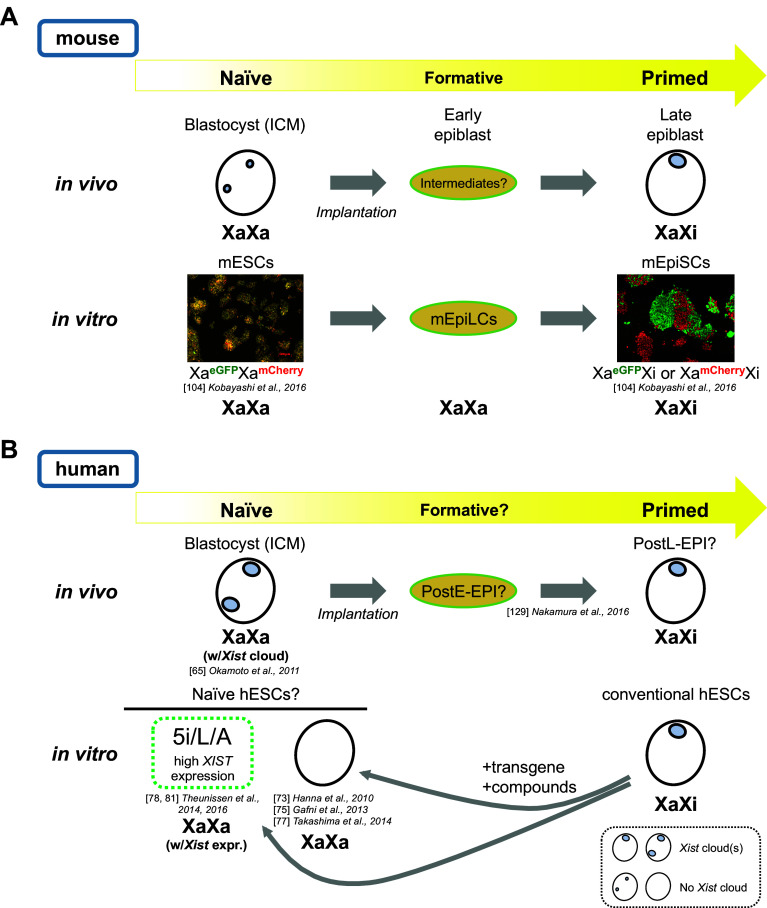
Relationship of naïve-to-primed transition and XCI states in mice and humans. **a** Schematics of the relationship between naïve and primed states and XCI in mice. XaXa represents two active Xs, while XaXi represents the presence of an Xi. In mice, the cells of the ICM of the blastocyst are thought to represent the naïve state in vivo. They exhibit two pinpoint *Xist* RNAFISH signals (tiny blue dots) inside the nucleus, which indicates that these cells have not initiated XCI. Upon differentiation, the cells likely go through multiple intermediate stages before becoming the late epiblast cells, which have acquired the primed state in vivo and exhibit a single *Xist* RNA cloud coating the Xi (large blue foci). The naïve state can be captured in vitro in the form of mESCs cultured in medium containing either serum/LIF or 2i/LIF, with the latter showing more uniform naïve properties. Female naïve mESCs exhibit active transcription from both Xs as shown by the uniform yellow fluorescence of female mESCs derived from the Momiji mice [104]. In the Momiji mice, the cells have a CAG promoter-driven *eGFP* reporter on one X and a *mCherry* reporter on the other at the same locus, and therefore the cells exhibit yellow fluorescence when the reporters are biallelically expressed, such as in naïve mESCs. The conversion of mESCs to mEpiSCs in vitro may occur via an intermediate stage represented by the ‘formative’ EpiLC state, which has not initiated the XCI and resemble the post-implantation epiblast (E5.75) based on transcriptome data [88]. The primed mEpiSCs derived from the Momiji mice show either green or red fluorescence, indicating that the cells have inactivated one of the two X chromosomes by random XCI. **b** Schematics of the relationship between naïve and primed states and XCI in humans. The schematic drawing is somewhat speculative, with areas of uncertainty indicated by several question marks. First, there are multiple ‘naïve’ hESCs derived from conventional hESCs by various methods in vitro with slightly different properties including the regulation of X*IST* lncRNA, which is highly expressed in the 5i/L/A culture condition [78] but not in others [73, 75, 77]. In human blastocysts, cells show biallelic expression of X-linked genes, indicating that they are in an XaXa state, but paradoxically exhibit double *XIST* RNA cloud accumulation per nuclei [65]. The precise relationship of these various ‘naive’ cells established in vitro and their relationship to the cells of the blastocyst in vivo are still unclear. Upon differentiation, the ICM cells presumably go through a series of intermediate states including those that represent the post-implantation early epiblast (postE-EPI) and late epiblast (postL-EPI), based on a recent study of the early embryogenesis of cynomolgus monkeys [129]

Although no clear chromatin differences between naïve and primed cells have been reported, female X-chromosome inactivation (XCI) state could be the epigenetic signature that best indicates the differences in developmental potential between these cell types, and how it changes during differentiation. While XCI is eventually completed in all somatic lineages following the post-implantation epiblast stage, the processes that lead to XCI are regulated in spatiotemporally distinct manners during early embryogenesis [45]. Female mEpiSCs have been shown to exhibit an Xi, as shown by the presence of a H3K27me3 focus [21, 29], whereas mESCs have two active X chromosomes (Xa), and the reprogramming of somatic cells to iPSCs is accompanied by reactivation of the Xi [18]. Forced expression of exogenous Klf4 in mEpiSCs also leads to Xi reactivation [21]. When one differentiates female miPSCs, XCI is initiated and the *Xist* long noncoding (lnc) RNA and H3K27me3 become enriched on the Xi in differentiating miPSCs [18, 21]. Thus, XCI state is closely linked to the cell’s differentiation state; naïve mESCs/miPSCs lack an Xi and primed mEpiSCs possess one (Fig. 1a).

**Fig. 2:**
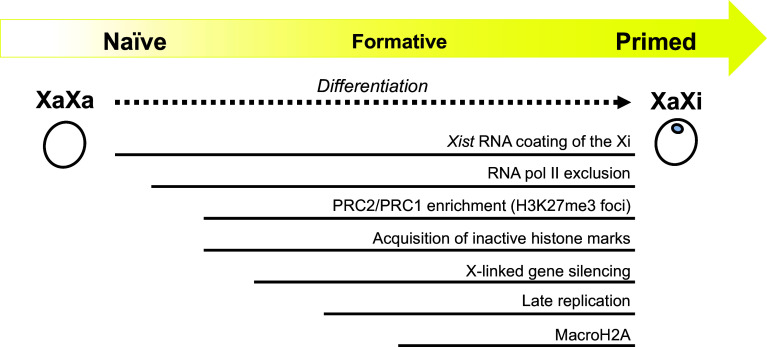
A rough outline of the temporal relationship of various epigenetic marks associated with the Xi based on mESC differentiation studies [46]. There are so many Xi-associated marks that have been and are being discovered that it is not entirely clear which set is conserved across species. It is also not clear which marks are present in primed and formative pluripotent cells

Many regulatory steps lead to the completion of XCI, and XCI states come in different flavors [25, 46] (Fig. 2). For instance, during mESC differentiation in vitro, it is believed that the coating of the future Xi by *Xist* RNA is one of the earliest events upon initiation of XCI. Afterward, the exclusion of RNA pol II and active histone modifications of the future Xi occur, followed by PRC2 and PRC1 recruitment [47, 48] and the addition of repressive histone marks, H3K27me3 and H3K9me2, to the Xi [49, 50]. Recruitment of macro-H2A and Ash2L are considered to be rather late events in XCI [51, 52], as is the chromosome-wide replication timing switch from the early to late S-phase of the Xi [53, 54]. The XCI mark used to define XCI in a given report thus warrants close attention. For instance, if H3K27me3 foci on the Xi are used as a mark to define XCI and were detected in a cell type analyzed, this does not guarantee that the Xi in this cell type had completed the XCI events downstream of H3K27me3 foci formation. It should also be noted that the order of events described above are based on analyses of cell populations during mESC differentiation [46], but there is no strong evidence of whether this order holds true in individual cells or during mouse embryogenesis.

Interestingly, we recently witnessed the discovery of a wide variety of *Xist* RNA-binding proteins, which together built a foundation for dissecting the earliest phase of heterochromatin formation on the Xi [55–59]. Meanwhile, the rise of Hi-C (a genome-wide, high-throughput chromosome conformation capture method) [60] and its application to XCI studies has led to the discovery that the Xi is subdivided into two ‘megadomains,’ which are several tens of megabases in size [57, 61, 62], as well as that TAD (topologically associating domain) boundaries disappear from the Xi upon XCI [63]. Thus, there are already many novel Xi-specific marks that are waiting to be characterized, and more will certainly be identified in the future. Where each of these marks lies in the context of the multi-step XCI process, their causal relationship, and how these marks relate to the primed state are important areas of future investigation in the effort to better understand the epigenetic state of the Xi in primed cells (Fig. 2).

## Conserved features of the X-chromosome inactivation process and the 3D genome organization

XCI regulation in mice is fairly complex, but in other species it is even more complicated [64]. Analyses of XCI in humans and rabbits have revealed that the XCI processes are markedly different between rodents and other eutherians. For example, unlike in mice, human *Xist* RNA is expressed from and coats both X chromosomes in females in the ICM of the early blastocyst, and this is maintained even in late blastocysts (Fig. 1b) [65]. Similarly, ~ 25% of early blastocyst cells in female rabbits show two *Xist* RNA clouds, although one of the two clouds disappears by the late blastocyst stage. Thus, the initial *Xist* coating of X chromosomes in human and rabbit ICM cells is unrelated to their future XCI fate. Meanwhile, human and rabbit female somatic cells exhibit a single Xi with an *Xist* cloud, meaning that the two *Xist* clouds on both X chromosomes eventually become localized only to the Xi at some point during development. How cells achieve this is currently unknown. Because lagomorphs (rabbits) are more closely related to rodents (mice) than any other mammals and yet show more similarity to primates (humans) with respect to XCI regulation, it could be argued that rodents are the exception rather than the rule [65]. These observations highlight the importance of understanding XCI regulatory processes in non-rodent organisms, and of distinguishing the processes that are conserved across species from those that are specific to certain organisms. Such comparative analysis would help us to identify the conserved core regulatory mechanisms of XCI and to elucidate conserved Xi-specific epigenetic signatures that demarcate primed from naïve states.

Interestingly, one of the most conserved properties of the Xi is its replication late in S-phase [54, 66, 67], which is observed not only in eutherians but also in marsupials and monotremes [68]. In female mESCs, the two X chromosomes show early replication, while in female mEpiSCs the Xi replicates late [69], consistent with the Xi becoming late replicating after the post-implantation epiblast stage in vivo [53]. DNA replication timing has recently been shown to be closely associated with the 3D genome organization [69–71], and whether or not the early to late replication timing shift of the Xi reflects a 3D organizational change is of interest. As discussed earlier, allele-specific Hi-C analysis has led to the elucidation of the 3D organization of the Xi, which exhibits two ‘megadomains’ and lacks TAD boundaries in somatic cells, such as fibroblasts, brain cells, and neural progenitor cells [57, 61–63, 72]. It remains unknown whether these novel properties of the Xi are observed in primed cells, and how they relate to the compact Xi structure, or the Barr body. Moreover, naïve and primed cells exhibit distinct enhancer usage for some genes, which suggests differences in long-range chromatin interaction [34]. Differences in the 3D genome organization between naïve and primed cells are an interesting yet relatively unexplored area and may provide us with clues into the broader implications of the Xi structure observed only in females.

## Naïve pluripotent state in human cells?

Conventional human ESCs often exhibit signs of XCI and high Oct4 PE enhancer activity, and because their culture condition is similar to mouse EpiSCs they have been regarded as primed cells [6]. Whether such a state as naïve hESCs exists is unclear [6], but attempts have been made to establish naïve hESCs to directly address whether or not the naïve state exists in human cells. By 2014, several groups had reported the establishment of ‘naïve’ hESCs [73–80]; these show gene expression profiles more closely resembling those of mESCs than the conventional hESCs, based on a clustering analysis of their transcriptomes [73–80]. Certain differences have been found between these ‘naïve’ hESCs and mESCs, however. For instance, two distinct cis-regulatory elements of Oct4, DE and PE, are active primarily in naïve and primed states, respectively, in mice and these various human ‘naïve’ cells do exhibit high DE activity. However, DE activity was often only transient and most ‘naïve’ cells eventually switched to activate PE upon long-term culture. The only exception was the 5i/L/A culture condition reported by Theunissen et al. [78]. However, 5i/L/A culture caused an erasure of DNA methylation in regions subject to genomic imprinting [81], which would be a problem in regenerative medicine. Furthermore, XCI states were variable among these ‘naïve’ cells, with some showing two *XIST* clouds on both X chromosomes, but no clouds in others (Fig. 1b). DNA methylation states of the *XIST* promoter also vary among ‘naïve’ cells, with some showing higher methylation levels than others. These issues have to be resolved in parallel with the elucidation of the conserved features of the XCI processes. In any case, the pursuit of a ‘gold standard’ method for maintaining naïve hESCs in culture is certain to continue [82].

Regarding hESCs, a peculiar phenomenon called erosion of XCI has been observed [83, 84]. On long-term culture of female hESCs, the XCI marks on the Xi, namely *XIST* cloud and H3K27me3, disappear and transcriptional activity is derepressed on the Xi [83]. When this XCI erosion occurs, *XACT* lncRNA, which normally coats the Xa, covers the Xi prior to it losing its *XIST* RNA coating [85, 86]. This is particularly troublesome, as once hESCs/hiPSCs experience XCI erosion, they never undergo XCI again, even if they are differentiated [86]. This is clearly distinct from the Xi reactivation phenomenon that occurs during reprogramming, and poses a safety issue when hESCs/hiPSCs are used as source cells in the development of regenerative medicine. We clearly need to accumulate more knowledge on culture conditions that can stably maintain epigenetic states of naïve or primed hESCs/hiPSCs in a controlled manner. For instance, a very recent study revealed that hiPSCs with an eroded Xi can still undergo XCI upon differentiation if the cells are converted to a ‘naïve’ state [78] prior to differentiation [87], implying that there are ways to reset the eroded state. These authors also revealed that ‘naïve’ hESCs derived from the blastocyst and from conventional (i.e., primed) hESCs (by 5iLAF culture similar to 5i/L/A [78] but with FGF2) both exhibited two active Xs and yet only the former exhibited one or two *XIST* RNA clouds [87]. Interestingly, ‘naïve’ hESCs derived from conventional hESCs formed an *XIST* RNA cloud on a single X after adaptation in 5iLAF for several passages, suggesting that they became more similar to ‘naïve’ hESCs directly derived from the blastocyst [87].

## New tools to approach and monitor naïve and primed pluripotency and their transitions

In human cells, it is difficult to describe the epigenetic differences between naïve and primed cells/states simply because, as discussed earlier, the human naïve state is not fully understood. Many different ‘naïve’ human cells have been proposed, and at present it is impossible to say which one corresponds to the naïve state in mice (Fig. 1b). Addressing this is important not only for human stem cell biology, but also for the understanding of evolutionarily conserved aspects of the epigenetic differences between naïve and primed cells. Another important challenge is to understand the processes by which naïve cells acquire the primed pluripotent state during differentiation. However, several research advances and new technologies have been reported in this area.

First, a distinct type of stem cells named EpiLCs (epiblast-like cells) was successfully derived from mESC differentiation in vitro [88]. Although it has not been possible to stably maintain EpiLCs, they can be generated reproducibly and relatively easily by differentiating naïve mESCs for 2 days in adherent culture using defined medium conditions [8, 89]. Mouse EpiLCs exhibit gene expression patterns that closely resemble the early epiblast in vivo and serve as an excellent substrate to generate primordial germ cell-like cells (PGCLC) in vitro, while mESCs and mEpiSCs do not [88, 90]. Moreover, female mouse EpiLCs lack H3K27me3 enrichment on the future Xi, which indicates that XCI either has not initiated or at least is far from completion in these cells [90] (Fig. 1a). Thus, mouse EpiLCs represent a unique differentiation stage somewhere in between the naïve and primed states; their gene expression profile has clearly shifted from a mESC-like to an epiblast-like state and resemble the early primitive ectoderm-like (EPL) cells [91] or Rex1-negative mESCs [92], but their epigenetic state is still closer to the mESCs. The EpiLC state was recently designated the formative pluripotency state [93], and EpiLCs provide the unique opportunity to scrutinize known properties of naïve and primed states and see when they change during naïve–formative–primed transitions or whether they are exclusively associated with the naïve or primed states [89, 94–97].

*Rex1/Zfp42* is one of the representative ICM genes that are sharply downregulated upon exit from naïve state during mESC differentiation. Austin Smith’s group generated a mESC line in which a transgene encoding GFP with a half-life of 2 h was knocked into the *Rex1* locus [98]. When cultured in the 2i condition, mESCs were Rex1-positive, whereas in serum/LIF medium without 2i, GFP (Rex1)-positive and negative cell populations coexisted, allowing them to focus on the earliest phase of differentiation in which the mESCs exit the naïve state [99]. This system, combined with haploid mESCs [100–102], which are an excellent tool for forward genetics, was used to screen for factors required for the cells to exit the naïve state [99].

Recently, Choi et al. generated mESCs derived from Oct4-ΔPE-GFP and Oct4-ΔDE-RFP double transgenic mice [103]. In mouse embryos, the cells were GFP positive (i.e., DE positive) until the blastocyst stage and became GFP/RFP double positive after E5.5 (i.e., DE and PE positive), whereas in mESCs cultured in vitro, the cells were GFP positive in 2i/LIF condition and GFP/RFP double positive in serum/LIF. Interestingly, Choi et al.’s mEpiLCs derived from these mESCs became RFP positive [103]. However, their EpiLCs, designated as EpiSC-like cells, went through multiple passages and were clearly not the equivalent of the EpiLCs described by Hayashi et al. [88]. It will be interesting to see whether the EpiLCs reported by Hayashi et al. utilize Oct4-DE, -PE, or both regulatory elements.

Kobayashi et al. recently established a novel X-linked eGFP/mCherry dual reporter mouse strain, named Momiji (named after the autumn leaves of Japanese maple trees), which enables real-time monitoring of the XCI state during mouse development [104]. The first mice with an X-linked GFP reporter, X-GFP, were established by Hadjantonakis et al. [105]. These X-GFP mice were used to establish GFP-negative EpiSCs, which, when cultured in LIF+ medium for a few weeks, generated GFP-positive cells, providing a rare example of spontaneous primed-to-naïve conversion [29]. In Momiji mice, CAG promoter-driven *eGFP* and *mCherry* are knocked into the maternal and paternal *Hprt* locus, respectively (or vice versa in reciprocal mice) [104], which allows simultaneous monitoring of both X chromosomes. The same system was built into the *Pgk1* locus as well. In either case, green or red indicates random XCI, while yellow indicates the presence of two active Xs in ESCs or upon Xi reactivation (Fig. 1a). How well *Hprt* and *Pgk1* loci represent the chromosome-wide transcriptional activity of the entire X chromosomes is a matter of debate, but with the Momiji mice the XCI state can now be monitored live in early mouse embryogenesis and during mESC differentiation in vitro [104]. How the changes in the XCI state relate to the cell fate transitions during mouse embryogenesis is a challenge for the future that could be addressed with this system.

## Future directions: new approaches, single-cell epigenomics, and live-cell imaging

A growing body of evidence suggests that there must be a set of epigenetic marks, both known and unknown, that contributes to the crucial difference between naïve and primed pluripotent cells, but we are still only halfway through the journey to understanding. We still need to precisely describe various epigenetic events and clarify their causal and temporal relationships one by one. In doing so, at least three important issues come to mind.

First, we were interested to note that many of the studies reviewed in preparing this article relied on a limited number of markers when distinguishing between naïve and primed states. In general, there may be an over-reliance on the differential usage of Oct4-DE and -PE, which is merely a single gene regulatory event. Moreover, the response of these enhancers is clearly not all-or-none, indicating the importance for future studies of cautiously determining the pluripotency state by examining additional features. This trend may be a reflection of how little is known about the epigenetic differences between naïve and primed states. The field should continue to search for additional reliable markers that can clearly distinguish the two states.

In this regard, one emerging area of interest is the role of energy metabolism in regulating the epigenetic status of naïve and primed cells [106, 107]. In a seminal study, Zhou et al. reported that naïve mESCs rely on both anaerobic (glycolytic) and aerobic (mitochondrial) respiration, while primed mEpiSCs rely almost exclusively on glycolysis [108]. Importantly, this metabolic difference is observed in vivo in the context of the transition from the ICM of the mouse blastocyst to the post-implantation epiblast [108], as well as conserved in the context of naïve vs. primed human ESCs/iPSCs [77, 109]. These observations provoked interest in the potential roles of various metabolites in regulating the epigenetic states in naïve and primed cells and led to, for instance, the discovery of the role of α-ketoglutarate, a TCA (tricarboxylic acid) cycle intermediate, in maintaining naïve pluripotency through promoting histone/DNA demethylation [110], while accelerating differentiation of primed mouse EpiSCs and human ESCs [106]. Nicotinamide *N*-methyltransferase (NNMT), which controls the amount of S-adenosyl methionine (SAM) available for H3K27me3, is required to maintain low H3K27me3 levels and keeps the Wnt pathway active and the HIF pathway inactive, helping hESCs to sustain their ‘naïve’ state [109]. Maintenance of a constant SAM level in contrast is crucial to the self-renewal of human ESCs/iPSCs [111].

Secondly, we will need a good in vitro ESC differentiation system that is homogeneous and synchronous, which should help in elucidating the order of various events that eventually lead to the formation of primed cells originating from naïve cells, and enables next-generation sequencing (NGS)-based epigenomic analyses, even with cell populations. In recent years, many excellent ESC/iPSC differentiation protocols have been developed to reconstitute certain lineage differentiations in vitro and generate tissue organoids. Many of these protocols rely on insights obtained from basic developmental biology over the years to recapitulate germ layer and tissue differentiation in a stepwise manner in vitro [90, 112, 113]. The earliest steps of these sophisticated protocols may yield clues on improving early differentiation processes relevant to the naïve-primed transition, which was exactly the case with EpiLCs and PGC development [88].

In addition, continuous efforts to improve ESC culture conditions may also be important. For example, Nichols and Smith first proposed that the epiblast in vivo constitutes the ‘ground state,’ meaning a fully unrestricted population that harbors the requisite developmental potency and flexibility to produce all embryonic lineages [5], and the term ‘ground state’ has also been used to describe the developmental state of naïve mESCs cultured in 2i/LIF medium in vitro [7]. However, Yagi et al. recently reported that DNA methylation imprints are erased in female mESCs grown in 2i/LIF and these cells also exhibited impaired autonomous embryonic and placental development as assayed by tetraploid embryo complementation or somatic cell nuclear transfer [38]. This warrants reconsideration of the definition of ‘ground state’ pluripotency in vitro and underscores the potential of the ‘alternative 2i (a2i)’ approach with the Mek1/2 inhibitor replaced by a Src inhibitor CGP77675, which can preserve the epigenetic stability of genomic imprints and the developmental potential of early passage female mESCs [38]. It should be noted that the a2i approach is not perfect and problems can arise upon prolonged culture of female mESCs, but this approach should certainly stimulate the field.

The third key issue is in vivo analysis. Once differential properties between naïve and primed cells are identified, it is essential to address whether those differences are also observable in vivo. DNA methylation is a classic example in which behaviors in the dish differ from those in the body [41]. Moreover, as in the case of XCI, the more embryonic tissues and species analyzed, the more unambiguous the distinction will be between conserved and species-specific properties of naïve and primed states. In general, however, in vivo analysis is challenging. Conventional cell-based assays that utilize fluorescent labeling such as immunostaining and FISH-based approaches are feasible, but to perform time-course analyses some form of a live imaging system is preferred. These methods do not allow visualization of genome-scale properties, while single-cell NGS approaches do. Single-cell epigenome profiling by NGS, however, is still challenging. Many single-cell epigenome profiling methods have been reported, e.g., for histone modifications [114], DNA methylation [115, 116], ATAC-seq [117], Hi-C [118–120], Dam-ID [121], and so on [122], but only a few have been applied to the analysis of embryonic cells in vivo due to various issues, including cost, resolution, and technical difficulties [120, 123–125]. Serious efforts are now being made, but there are still few single-cell, NGS-based epigenome profiling methods that are sufficiently reliable for use in the analysis of embryonic cells in vivo.

However, the situation is different for RNA-seq analyses. Various single-cell RNA-seq protocols have been established that are practical and reliable enough to be applied to the analysis of embryonic cells in vivo. Furthermore, the analytical platform has gradually shifted from multi-well plates to microfluidics to droplet-based technologies, which will lead to new and important discoveries regarding the behaviors and properties of single cells within large cell populations [126–128]. The timing at which chromosome-wide silencing of the Xi takes place in mice, or the switch from imprinted to random XCI during the morula–blastocyst–epiblast transition, may be amenable to single-cell RNA-seq analysis. A recent single-cell RNA-seq study of early embryogenesis in the cynomolgus monkeys has demonstrated the power of in vivo analysis and built a foundation for discovering conserved features of the naïve-to-primed transition in vivo [129] (Fig. 1b). Furthermore, the use of cynomolgus monkeys allows us to test the ability of cells to contribute to chimeric animals, which should help in revealing the conserved features of the naïve and primed states [130].

These novel approaches and their future developments, combined with steady efforts to elucidate the causal and temporal relationships between different properties of naïve and primed cells, should gradually reveal their critical intrinsic differences. Moreover, such efforts may lead to the identification or in vitro capture of additional intermediate states in between naïve and primed, perhaps akin to the manner in which the EpiLCs were identified. Through these efforts, the next frontier in this field should emerge.
